# Does Fermentation Really Increase the Phenolic Content in Cereals? A Study on Millet

**DOI:** 10.3390/foods9030303

**Published:** 2020-03-07

**Authors:** Diletta Balli, Maria Bellumori, Laura Pucci, Morena Gabriele, Vincenzo Longo, Paolo Paoli, Fabrizio Melani, Nadia Mulinacci, Marzia Innocenti

**Affiliations:** 1 Department of NEUROFARBA, Nutraceutical section, University of Florence, Via Ugo Schiff 6, 50019 Sesto Fiorentino, Italy; diletta.balli@unifi.it (D.B.); maria.bellumori@unifi.it (M.B.); fabrizio.melani@unifi.it (F.M.); marzia.innocenti@unifi.it (M.I.); 2 Institute of Agricultural Biology and Biotechnology, National Council of Research, CNR, Via Moruzzi 1, 56124 Pisa, Italy; pucci@ibba.cnr.it (L.P.); gabriele@ibba.cnr.it (M.G.); v.longo@ibba.cnr.it (V.L.); 3 Department of Experimental and Clinical Biomedical Sciences “Mario Serio”, University of Florence, Viale Morgagni 50, 50134 Firenze, Italy; paolo.paoli@unifi.it

**Keywords:** fermented millet, *Lactobacilli*, flavonoids, cinnamic acids, PTP1B enzyme, molecular dynamic

## Abstract

Millet is underutilized in Europe, despite its advantages compared to other common cereals. In Asia and Africa, millet is mainly eaten in fermented form; its consumption has beneficial properties on human health. Three millet batches were compared in terms of free and bound phenols by High Performance Liquid Chromatography-Diode Array Detector-Mass Spectrometry (HPLC-DAD-MS). The richest one in terms of bound phenols was selected for testing via a basic (0.1 M NaOH) and an acidic (1.2 M H_2_SO_4_) hydrolysis, in which 149.3 and 193.6 mg/100 g of phenols were recovered, respectively. The ability of fermentation, with yeast and *Lactobacilli*, to increase the content of phenolic compounds was verified. Five withdrawalswere performed to verify the influence of fermentation time on the total phenolic content. The greatest phenolic content was observed after 72 h. Fermentation increased the cinnamic acids and flavonoids contents by approximately 30%. Vitexin and vitexin 2″-*O*-rhamnoside contents were significantly higher in the fermented millet; these compounds partially inhibit the protein tyrosine phosphatase enzyme, which is overexpressed in type-2 diabetes. A molecular dynamic simulation showed the two flavonoids to be allosteric inhibitors. The phenolic extract from fermented millet demonstrated a higher level of antioxidant protection on human erythrocytes by ex vivo cellular antioxidant activity in red blood cells. In this context, functional foods based on fermented millet could represent a new trend in European markets.

## 1. Introduction

Millet is a generic term that includes various small grains belonging to the *Poaceae* family. It exhibits great advantages compared to other cereals such as wheat. Millet is able to grow almost anywhere thanks to its resistance to drought, high temperature tolerance, and low incidence of mycotoxin contamination [1]. In 2016, millet was the sixth most produced cereal worldwide. Furthermore, it contains high levels of proteins, minerals, and vitamins, that confer upon it higher nutritional value than nonmillet cereals. Its proteins are reported to possess significant levels of essential amino acids. Furthermore, compared to other cereals, it is rich in slowly digestible and resistant starch, which are important for the prevention of diseases related to type-2 diabetes, obesity, and coronary heart disease, because of their resistance to digestion, resulting in the slow release of glucose into the blood stream and reducing postprandial glycaemic and insulinemic response [2]. In light of all these characteristics, the cereal was recently defined as “nutritious millet” or as a “nutritious cereal”. Millet could be applied to formulate gluten free products, a growing field in the food industry because of the continuous increase in the prevalence of celiac disease (approximately 3% of the world population). Gluten free cereals have gained special consideration not only for their nutritional suitability for celiacs, but also for their properties linked to the presence of many phytochemicals which are able to act as preventive factors against several human diseases [3,4]. For millet, research related to the formulation of new gluten free foods is challenging. Despite the potential advantages of millet consumption, this cereal is poorly utilized in Europe by humans due to the scarce economic and technological support it has received [5]. In contrast, in African and Asian cuisines, millet is considered a staple. Approximately 90% of world production is destined for human consumption in the form of a fermented cereal [6]. Fermentation has been traditionally used for millennia as a cost-effective and low-energy preservation process to produce indigenous fermented foods with improved nutritional, health, and sensorial qualities [7,8]. In this context, the fermentation of cereal is an ancient technique which is economically sustainable, capable of increasing the concentration of beneficial substances and making proteins more available, reducing antinutritional components, providing food preservation, and improving food texture and shelf-life through the action of several microorganisms [9,10,11,12]. Cereal-based fermented products are an important part of diets in several African, south Asian, and middle eastern countries, constituting almost one-third of the diets there. The low cost and the need for only low-tech procedures which are accessible to poorer rural societies are two of the main advantages of fermentation [13,14].

A great deal of epidemiological evidence has highlighted how the consumption of cereal fermented foods, taken daily in high doses, has been associated with the reduced risk of cardiovascular disease, diabetes, and cancer [13]. This preventive effect has been associated with the presence of antioxidant molecules which are capable of reducing the amount of free radicals in vivo [15]. These natural antioxidants, such as phenolic compounds, are mainly present in bound form (conjugates with sugars, fatty acids, or proteins) in the nonfermented cereal. Acidic and alkaline hydrolysis need to be applied to allow the phenol release at the laboratory scale to evaluate the antioxidant levels of the grains. In contrast, cereal fermentation seems to be a suitable technique to enhance the release of bound phenols before consumption [16]. In particular, fermentation is reported to increase the bio-conversion of phenolic compounds from their linked or conjugated forms to their free ones, resulting in an increased concentration of phenolic components with greater antioxidant power [17]. The temperature of the process and the types of microorganisms used play a crucial role in increasing the antioxidant potential; they are responsible for the chemical transformations and for obtaining an optimal pH through the degradation of the cell membrane and release of bound phenolic compounds. Mimicking the Asian and African tradition, novel fermented nonwheat cereals could represent a promising trend in the production of new, healthy foods [18]. Today’s growing emphasis on a healthy and balanced diet has created a demand for new functional foods which are able to prevent the risk of several pathologies such as diabetes. Indeed, a lower incidence of type-2 diabetes was recognized in the Chinese population that consumed millet on a daily basis [19]. One of the possible mechanisms of action, linked to the reduction of diabetes, is the inhibition of protein tyrosine phosphatase 1B enzyme (PTP1B), which is overexpressed in type-2 diabetes. In silico studies on the PTP1B enzyme allowed us to better understand the interaction between the active sites of the enzyme and its target molecules. As far as allosteric inhibitors, thanks to molecular dynamic (MD) studies, it is known that the flexibility of WPD-Loop in the PTP1B enzyme is reduced by approximately 10% after interaction with a ligand [20,21]. Subsequently, the presence of an allosteric inhibitor was shown to reduce the mobility of the WPD-Loop and S-Loop [22]. Several natural flavonoids have been recognized as allosteric inhibitors of this enzyme [23].

The objectives of this work were: (a) to verify how a fermentation process performed with a mixture of yeast and *Lactobacilli* used for bread production influenced the phenolic profile in millet; (b) to measure the inhibition power of the millet extracts and their flavonoids on PTP1B enzyme; (c) to investigate on the mechanism of action of vitexin and vitexin 2″-*O*-rhamnoside through an in silico study; (d) to evaluate the antioxidant activity (CAA-RBC) of fermented and unfermented samples. The work aims to show how as a simple fermentation method can improve the nutritional and functional value of millet, a cereal not commonly used in Europe, paving the way for the design of new fermented products.

## 2. Materials and Methods

### 2.1. Samples and Reagents

Three batches of millet, namely M1, M2 and M3, were purchased from markets in Burkina Faso. Morphological evaluation, based on sample caryopses, suggested that the purchased millet was pearl millet, i.e., *Pennisetum glaucum* L. All solvents used were of analytical HPLC grade and were purchased from Sigma Aldrich (St. Louis, MO, USA). Water was ultrapure (Milli-Q^®^), ferulic acid (purity ≥ 99%), vitexin and vitexin 2″-*O*-rhamnoside (purity ≥ 95%) were from Extrasynthese (Genay, France). Phosphate buffer saline (PBS) tablets, 2,2′-azobis-(2-amidinopropane) dihydrochloride (AAPH), 2′,7′-dichlorofluorescein diacetate (DCFH-DA) and 6-hydroxy-2,5,7,8-tetramethylchromane-2-carboxylic acid (Trolox) were purchased from Fluka-Sigma-Aldrich, Inc. (St. Louis, MO, USA).

Human recombinant PTP-1B, expressed in *Escherichia coli* TB1 strain, and purified, and p-nitrophenyl phosphate, were purchased from Santa Cruz Biotechnology Inc. (Dallas, TX, USA).

### 2.2. Fermentation Process

The fermentation of the M1 and M2 samples (FM1 and FM2) was performed at the CNR of Pisa using a natural sourdough constituting a mixture of *Lactobacilli*, mainly *L. sanfranciscensis* and *L. pentosus*, and yeast strains in a ratio of approximately 100:1. Millet flour was obtained by grinding millet grains using a laboratory mill (M20 Universal mill IKA-Werke, Staufen im Breisga, Germany). Water (60%) was added to moisten the mixture, and then the sourdough, provided by “Lievitamente s.n.c.” (Viareggio (LU), Italy), was added to initiate fermentation. Once the product was fermented, it was frozen and freeze dried for two days with a Lyovac GT 2. The fermentation temperature was maintained at around 38 °C, while the pH reached a value of 4. Different withdrawals were performed at time 0 (T0) and during the process, that is, after 24 h (T1), 48 h (T2), 72 h (T3), and 96 h (T4) of fermentation.

### 2.3. Extraction of Phenolic Compounds

Free phenols. All the unfermented and fermented samples were treated with aqueous ethanol as previously described in the literature [24]. Briefly, the flour obtained from three milled samples (M1, M2, M3) was defatted twice with hexane (1:10 (*w*/*v*)) and kept on a mechanical shaker for 1 h. Then, 250 mg of millet flour was then extracted with ethanol:water 80:20 *v*/*v* under magnetic stirring in an ultrasound bath for about 15 min. The extract was centrifuged at 5000 rpm for 10 min, 10 mL of the supernatant were evaporated to dryness, and the residue redissolved in 1.5 mL of acidified H_2_O (1% HCOOH).

Total phenols. The flour of the three millet samples, and the fermented sample from M1 FM1-T3 obtained after 72 h were treated with the following hydrolytic procedure. The basic hydrolysis was carried out with the aid of ultrasound, as previously described [24]; 1 g of flour was treated with 25 mL of NaOH 0.1 M in MeOH/H_2_O 7:3 *v*/*v* and sonicated (40 MHz) for 1 h at 60 °C; the pH was neutralized with acetic acid, the suspension centrifuged at 5000 rpm for 10 min, and the supernatant was recovered (Method B). The samples of M1 and M2 with the corresponding fermented products after 72 h of fermentation (FM1-T3 and FM2-T3) were also treated with an optimized acidic hydrolysis for the recovery of total phenols by applying only a single extractive step according to the method described by Balli et al. [25]. Briefly, 1 g of defatted flour was suspended in 25 mL of acidified MeOH (1.20 M by H_2_SO_4_), the solution was sonicated for 180 min at 55 °C, and the sample was then centrifuged at 5000 rpm for 10 min (Method A).

### 2.4. Analytical HPLC-DAD

The millet extracts recovered after centrifugation were analyzed using a HP 1200L liquid chromatography equipped with a DAD detector (Agilent Technologies, Palo Alto, CA, USA). The column was a Poroshell 120, EC-C18 (150 × 3 mm, 2.7 µm, Agilent, Santa Clara, CA, USA) and was thermostated at 28 °C. The solvents for the mobile phase were (A) CH_3_CN and (B) 0.1% formic acid/water; the multi-step linear solvent gradient was the same as that proposed by Balli et al. [24]. Briefly, the multistep linear solvent gradient was 0–5 min: 0–10% A; 5–10 min: 10–15% A; 10–20 min: 15–30% A; 20–25 min: 30–35% A; 25–28 min: 35–40% A; 28–31 min: 40–45% A; 31–42 min: 100% A; 42–47 min: 100–0% A; equilibration time 5 min; flow rate 0.4 mL min^−^1; injection volume 10 μL. The following wavelengths were simultaneously detected: 240 nm, 280 nm, 330 nm, and 350 nm.

### 2.5. MS Analysis

The HPLC-DAD-MS analyses of the phenolic extracts were performed according to a method described by Balli et al. [25]. The HPLC-DAD-MS system was from Waters, and comprised a 2695 HPLC, 2996 PAD, and 4 microtm MS equipped with Zspray ESI source. The ESI interface parameters were capillary 2.90 kV, cone 64 V in the first 18 min and 30 V until the end of the analysis, source temperature 120 °C, desolvation temperature 350 °C, cone gas flow 19 (L/Hr), and desolvation gas flow 350 (L/Hr). Data were acquired in negative ion mode from 110 *m*/*z* to 1000 *m*/*z*.

### 2.6. Quantitative Determination of Phenolic Acids And Flavonoids

The phenolic acids were evaluated using a five-point calibration curve of ferulic acid at 330 nm, (R^2^ = 1, linearity range 0–0.21 µg); the content of flavonoid aglycones was determined using a five-point calibration curve with vitexin (purity ≥ 95%) at 350 nm, linearity range 0–0.21 µg (R^2^ = 1.0); vitexin 2”-*O*-rhamnoside (purity ≥ 95%) at 350 nm, linearity range 0–0.11 µg, (R^2^ = 1.0) was selected to quantify the glycosylated flavonoids.

### 2.7. Folin-Ciocalteu Reducing Capacity

Polyphenols, estimated as Folin-Ciocalteu (FC) reducing capacity, were determined as reported by Gabriele et al. [26] and expressed as mg/100 g dry weight (DW).

### 2.8. Inhibition Test on PTP1B Enzyme

The two flavonoids, vitexin and vitexin 2″-*O*-rhamnoside, were tested on the PTP1B enzyme at concentrations of 0.205 mg/mL and 0.111 mg/mL, respectively. Enzymatic assays were carried out using human recombinant PTP1B and p-nitrophenylphosphate (pNPP) as reference substrate [27]. Briefly, the pNPP (2.5 mM final concentration) was dissolved in sodium β,β-dimethyl glutarate buffer (75 mM, pH 7.0), containing 1mM ethylenediaminetetraacetic acid (EDTA) and 1 mM dithiothreitol. Reactions were started by the addition of aliquots of the enzyme and stopped by adding 2 mL of KOH 0.2 M. The released *p*-nitrophenolate was quantified by measuring the absorbance of the final solution at 400 nm (ε = 18,000 M^−1^ cm^−1^). The net hydrolysis rate was determined by subtracting the value of spontaneous hydrolysis rate of pNPP from each sample. The inhibitory power of vitexin standards was tested by adding different amounts of standard solutions, i.e., 5, 10, and 20 µL/mL for vitexin and 12, 25, and 50 µL/mL for vitexin 2″-*O*-rhamnoside. Furthermore, the two flavonoids mixed together (MIX-std) were tested in a concentration of 0.082 mg/mL and 0.111 mg/mL for vitexin and vitexin 2″-*O*-rhamnoside, respectively, adding 50 µL/mL. In addition, the aqueous extracts of unfermented (M1) and fermented flour (FM1) in a concentration of 1g/mL were both tested by adding 60 and 20 µL/mL, respectively. The percentage of inhibition was calculated by normalizing the absorbance values obtained for assays carried out in the presence of the inhibitor versus the control test. All the results were expressed as a mean of three independent experiments.

### 2.9. Molecular Dynamics Simulation for PTP1B Enzyme

A 10 ns MD simulation was performed for all the complexes using the GROMACS v5.1 program [28]. The partial atomic charge of the structures was calculated with CHIMERA [29] using the AM1-BCC method, and the topology was created with ACPYPE [30] based on the routine Antechamber [31]. The OPLS-AA/L all-atom force field [32] parameters were applied to all the structures. The simulation was performed in a vacuum. To remove bad contacts, energy minimization was performed using the steepest descent algorithm until convergence was achieved, or for a maximum of 50,000 steps. In the consecutive steps, equilibrations of the system were conducted in two phases: (1) canonical Particle Number, Volume, Temperature (constant) (NVT) ensemble, a 100 ps position-restrained of molecules at 300 K was carried out using a Temperature coupling thermostat (velocity rescaling with a stochastic term) to ensure the proper stabilization of the temperature [33]; (2) isothermalisobaric Particle Number, Pressure, Temperature (constant) (NPT) ensemble, a 100 ps position restrained of molecules at 300 K and 1 bar was carried out without using barostat pressure coupling to stabilize the system. These were followed by a 10 ns MD run at 300 K with position restraints for all protein atoms. The Lincs algorithm [34] was used for bond constraints to maintain rigid bond lengths. The initial velocity was randomly assigned according to Maxwell–Boltzman distribution at 300 K and computed with a time step of 2 fs; the coordinates were recorded every 0.1 ns.

### 2.10. Ex Vivo Cellular Antioxidant Activity (CAA-RBC) Assay in Red Blood Cells

Human blood samples from healthy volunteers were collected in EDTA-treated tubes and centrifuged for 10 min at 2300× *g* at 4 °C. Plasma and buffy coat were discarded and erythrocytes were washed twice with PBS pH 7.4. Fermented and unfermented millet samples were extracted with 10% of dimethyl sulfoxide in distilled water according to a method described by Gabriele et al. [35]. Briefly, the samples were centrifuged for 10min at 2300 g at 4 °C in a Jouan CR3i centrifuge (Newport Pagnell, UK), and the supernatant was collected, filtered on 0.2 µm cellulose filter (VWR International PBI, Milan, IT), and kept at 4 °C in the dark. The antioxidant activity of millet extract was tested at three different concentrations (0.01, 0.1 and 1 mg/mL) on human erythrocytes as described by Frassinetti et al. [36]. Trolox was used as a reference standard. The fluorescence was read at 485 nm excitation and 535 nm emission using a Victor TM X3 Multilabel Plate Reader (Waltham, MA, USA), and each value was expressed using the following formula: CAA unit = 100 − (∫SA/∫CA) × 100, where ∫SA is the integrated area of the sample curve and ∫CA is the integrated area of the control curve. Data are displayed as the average value of independent experiments performed on three different subjects. Blood samples were collected from healthy donors according to the regulations of “Fondazione G. Monasterio CNR-Regione Toscana” for the use of residual blood for research purposes. Informed consent was obtained from all participants and/or their legal guardians.

### 2.11. Statistical Analysis

Each experiment was performed in triplicate, and the results were expressed as the mean values ± SD; the EXCEL software in-house routines were applied. Differences between fermented and unfermented millet effects on human erythrocytes were analyzed by one-way analysis of variance (ANOVA) with Dunnett’s multiple comparison test and by unpaired t-test. A *p*-value lower than 0.05 was considered as statistically significant.

### 2.12. Proximate Analysis

Dietary fiber (soluble and insoluble) content was determined according to the AOAC Method 991.43 (determination of soluble, insoluble, and total dietary fiber in foods and food products; final approval: 1991).

## 3. Results and Discussion

### 3.1. Phenolic Characterization of Nonfermented Flours

**Figure 1:**
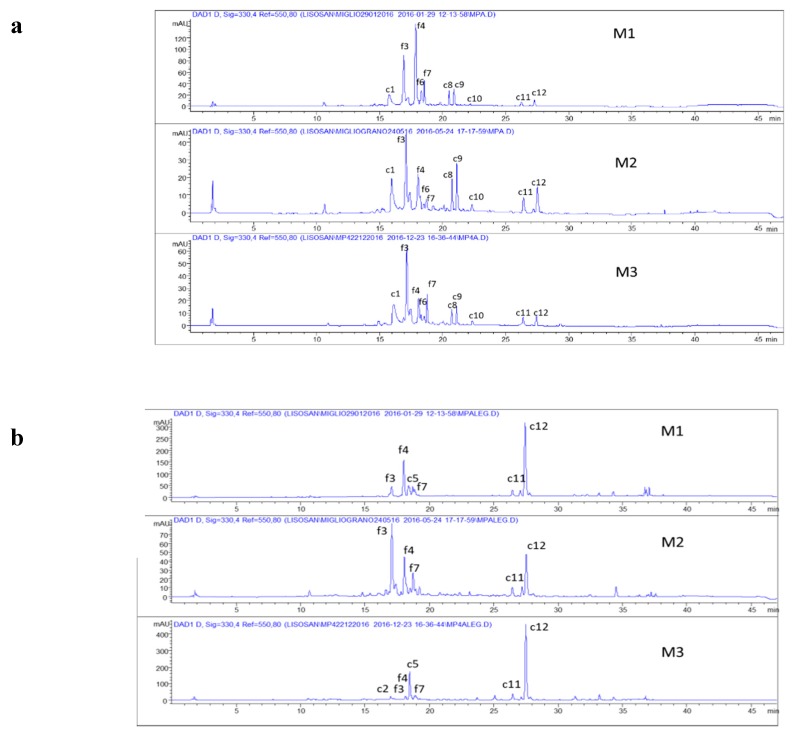
Chromatographic profiles at 330 nm of the free phenols (**a**) and total phenols obtained after basic hydrolysis (**b**) of the millet batches (M1, M2, M3). The identified compounds are listed in Table 1; c = cinnamic derivatives; f = flavonoids.

The HPLC profiles obtained from the extraction of free phenols from the three analyzed batches were very similar from a qualitative point of view (Figure 1), with compounds **3**, **4**, **6**, and **7** being recognized as flavonoids (f), and compounds **1**, **5**, and **8**–**12** as cinnamic (c) derivatives.

All the molecules listed in Table 1 and recognized by HPLC-DAD and MS analysis have already been identified in millet [25]. Chromatographic profiles showed differences in relative abundances among the three batches, while with regard to cinnamic derivatives, compound **c5**, identified as ferulic acid, was only present in M1 and M3 (Figure 1b). The extraction of total phenols was carried out on the whole defatted flour with a basic hydrolysis by NaOH 0.1 M. The applied hydrolytic conditions were milder than those proposed in the literature [19,37].

From a quantitative point of view, M1 showed total free phenols content of 127.4 mg/100 g, 87% of which were flavonoids; M2 showed a total of 48.2 mg/100 g with 68% flavonoids, and M3 showed a total of 64.0 mg/100 g with 83% flavonoids (Table 2).

Table 2 shows M1 to be the richest in terms of total phenols (149.3 mg/100 g), while for bound phenols, calculated as a difference between total and free phenols, M3 was the richest one (59.7 mg/100 g). To better evaluate the amount of bound phenols, M1 was also treated with an acidic hydrolysis which was recently optimized on a millet sample by the Response Surface Methodology according to Balli et al. [25]. Acidic hydrolysis guaranteed a recovery of the same phenols detected after the basic procedure but in a higher amount, i.e., 193.6 mg/100 g instead of 149.3 mg/100 g (Table 3).

Our results confirm that flavonoids in millet are almost completely present in free forms, in agreement with previous findings [25]; this makes them more stable in acidic conditions, with 111.6 mg/100 g recovered flavonoids compared to 71.9 mg/100 g obtained in a basic medium. Analogously, the acidic hydrolysis guaranteed a higher release of cinnamic derivatives bound to cell wall structures, reaching 82 mg/100 g.

### 3.2. Phenolic Characterization of Fermented Flours

Millet is usually decorticated before consumption to improve its edible properties, but this practice leads to a reduction in minerals, fibers, and antioxidants as phenolic compounds located in the peripheral parts of the grains [38]. Fermentation can be applied in order to treat the whole nondecorticated flour, thereby avoiding losing part of its functional components. Contradictory data about the effectiveness of fermentation in increasing phenolic content are present in the literature. Some studies reported that fermentation is able to increase the bio-conversion of phenolic compounds from their linked or conjugated forms to the corresponding free forms [17,39,40], while other works reported a decrease in the total phenolic content attributable to the rearrangement of some phenolic structures after a self-polymerization processes favored by the acidic conditions induced by fermentation [8,41].

**Figure 2:**
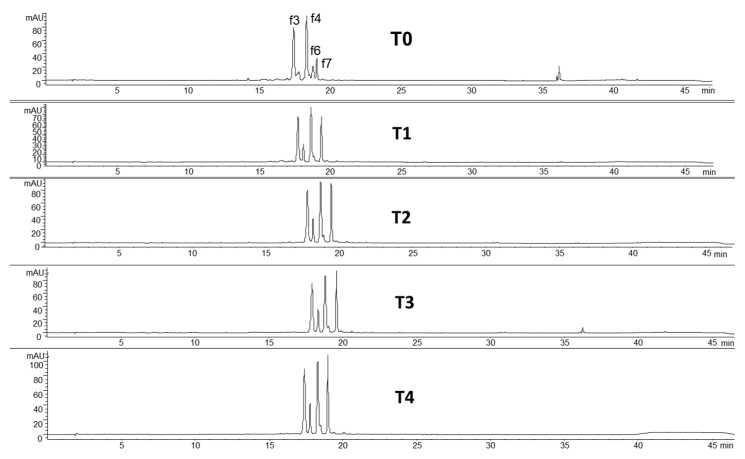
Chromatographic profiles at 350 nm of the fermented millet (FM1): at time 0 (T0), after 24 (T1), 48 (T2), 72 (T3) and 96 (T4) hours of fermentation. f3, luteolin (7-*O*-glucopyranosyl) 8-*C*-glucopyranoside; f4, vicenin II; f6, vitexin 2″-*O*-rhamnoside; f7, vitexin.

In this context, the use of HPLC-DAD and pure standards for the quantification of phenolic compounds before and after fermentation represents a more accurate method to estimate the real content. Herein, the extractions of both free and bound phenols were performed in order to verify whether fermentation was able to guarantee an almost complete release of the bound forms linked to the cellular structures, as cellulose or lignin. The M1 was selected to undergo the fermentation process. The HPLC-DAD profiles of free phenols from the fermented matrix showed a lower number of peaks compared to those obtained from the nonfermented sample; in particular, only flavonoids were detected (Figure 2). The absence of phenolic acids in the fermented flours could be attributable to the ability of *Lactobacilli* to metabolize some of the phenolic acids present in millet [42].

**Figure 3:**
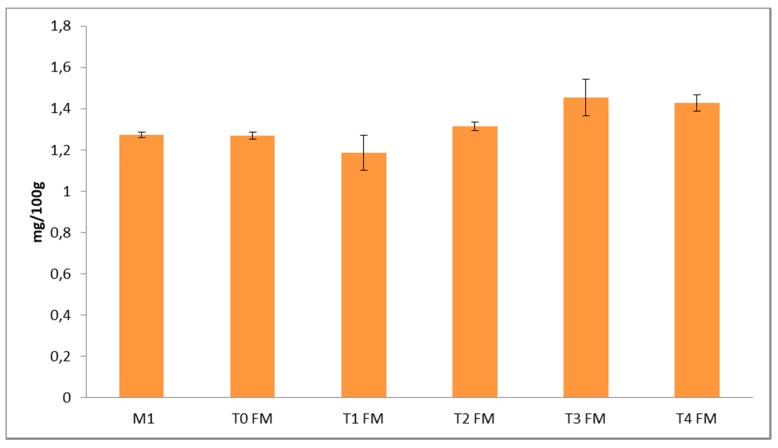
Free phenol content determined by HPLC-DAD in unfermented (M1) and fermented (FM1) millet flour at different withdrawals (T0-T4). Data are expressed in mg/100 g of dry weight as a mean of three different extractions.

The total content of free phenols changed during fermentation, passing from 126.9 mg/100 g at T0 to a maximum of 145.3 mg/100 g at T3; the amount after 96 h (T4) remained almost unchanged (Figure 3). According to this result, T3 was selected as the maximum time for fermentation.

The Folin Ciocalteu assay for FM1-T3 identified a significantly higher level of total phenols than in the M1 sample (270.1 ± 34.2 vs. 150.3 ± 8.1 mg GAE/100 g DW, *p* < 0.05). These results demonstrated an increase of phytochemical content after the fermentation process.

After the basic and acidic hydrolyses applied to the FM1-T3 sample, the presence was confirmed of the same phenolic compounds detected before fermentation. The basic hydrolysis allowed us to extract a higher amount of phenols (206.2 mg/100 g) compared to the basic hydrolysis of the unfermented sample (149.3 mg/100 g). Analogously, the acidic hydrolysis previously optimized for unfermented millet [25] was also performed on FM1-T3, again confirming the ability of this method to more efficiently extract the total phenols from cereals, obtaining 278.3 mg/100 g (Table 3). Even though a real comparison with the literature data should consider different environmental aspects, varieties and analytical methods, our results highlight that the total recovered phenols obtained by the acidic hydrolysis are in the higher range of those proposed in the literature, i.e., from 36.7 mg/100 g to 377.2 mg/100 g (Table S1).

**Figure 4:**
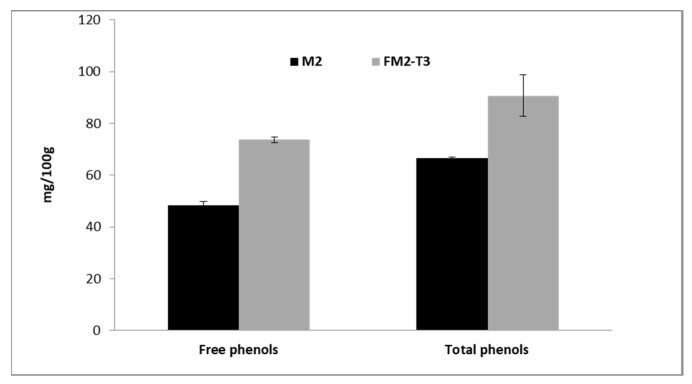
Free and total phenolic amount, by acidic hydrolysis, before and after fermentation in M2 sample. The fermented sample was withdrawn after 72 h of fermentation. Data are expressed in mg/100 g as a mean of three independent extraction.

The higher total phenolic values in the fermented sample could be attributed to the action of microorganisms that, using the soluble and fermentable fiber for their growth, released “mechanically trapped” phenolic compounds in the polymeric structures of the fiber. The evaluation of soluble fiber allowed us to verify that the M1 sample contained 2 g/100 g, which, after fermentation, strongly decreased to <0.10 g/100 g, demonstrating that fermentation was a useful treatment to release phenolic compounds which are mechanically or chemically trapped in the soluble fiber. For the M1 sample, the fermentation, induced an increase of up to 35% in the amount of these molecules with respect to their total content in unfermented flour. To confirm this result, another batch of millet (M2) was fermented using the same mixture of yeast and *Lactobacilli*, and free phenols and total phenols were evaluated after 72 h of fermentation by acidic hydrolysis. The results again highlighted an increase of 36% in the total phenolic content, passing from 66.5 mg/100 g to 90.7 mg/100 g for the unfermented and fermented matrix respectively (Figure 4).

### 3.3. Inhibition Test on PTP1B Enzyme

**Figure 5:**
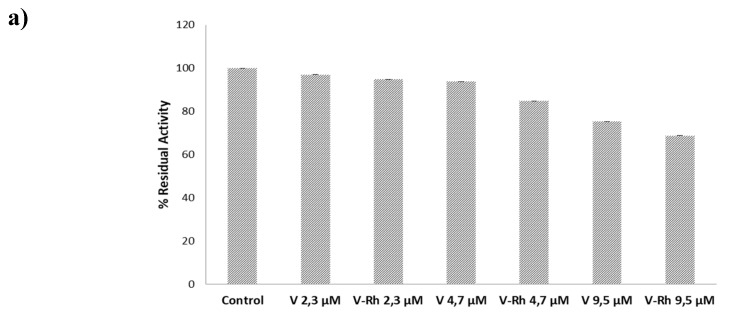

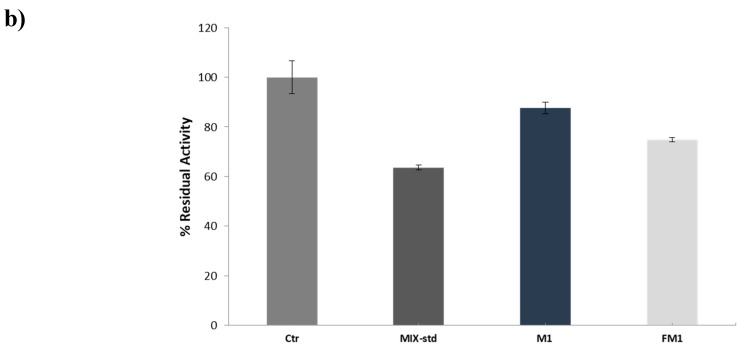
In vitro activity (as % of residual activity) of PTP1B enzyme. (**a**) vitexin (V) and vitexin 2″-*O*-rhamnoside (V-Rh), both tested at three concentration values: 2.3 µM, 4.7 µM and 9.5 µM. (**b**) comparison among a mixture of the two flavonoids (MIX-std) both at 9.5 µM and unfermented(M1) and fermented (FM1) extracts containing vitexin and vitexin 2″-*O*-rhamnoside at a concentration of approximately 9.5 µM, as mean value.

The two standards, i.e., vitexin and vitexin 2″-*O*-rhamnoside, were in vitro evaluated for their inhibitory effect on PTP1B, known as a negative regulator of insulin receptors. The choice of testing these flavonoids was made due to the inhibitory activity already shown by this class of molecules on the PTP1B enzyme [23]. In contrast, the cinnamic derivatives that were more abundant in millet were unable to inhibit the enzyme [43]. Previous literature has already reported the ability of vitexin to inhibit the PTPB1B enzyme at a concentration of 7.62 µM [44]. Considering the multiple structures of the previously used PTP1B enzyme, we decided to test the two standards of vitexin on our enzyme model. The results indicated that vitexin and vitexin 2″-*O*-rhamnoside were not able to inhibit the enzyme at concentrations of 2.3 µM and 4.7 µM, respectively, but an inhibition of 25% and 32% was identified for both of these flavonoids at 9.5 µM (Figure 5a). In light of this finding, the other three samples were tested: a mixture (MIX-std) of the two flavonoids at a concentration of 9.5 µM and the total unfermented extract (M1) and fermented extract (FM1) containing vitexin and vitexin 2″-*O*-rhamnoside, both at a concentration of approximately of 9.5 µM; see Figure 5b. The results highlighted inhibitory activity against the PTP1B enzyme of approximately 40% in the mixture extract (MIX-std), suggesting a sort of sum effect of the two flavonoids. Concerning the unfermented and fermented extracts (M1 and FM1), the results indicated an inhibition of 13% and 25%, respectively. These data suggest the presence, mainly in the unfermented flour, of other compounds that act as antagonists of the vitexin flavonoids, thereby reducing their activity on the PTP1B enzyme.

It is noteworthy that the concentrations of the two flavonoids in the fermented extract were 2–3 times higher than those needed to exert an enzymatic inhibition by 30%, i.e., 18 µM for vitexin and 22 µM for vitexin 2″-*O*-rhamnoside. Furthermore, the concentrations of the two flavonoids were higher in the fermented extracts (FM1-T3) with respect to the unfermented millet (MF1). After the recovery of total phenols in acidic condition, vitexin was 55% higher and vitexin 2”-*O*-rhamnoside was 60% higher. In light of these findings, we affirm that the daily consumption of fermented millet containing higher amounts of flavonoids with respect to unfermented flour could help to reduce the incidence of type-2 diabetes by the inhibition of the PTP1B enzyme, which is overexpressed in these subjects. Further analyses will be conducted to better understand whether the entire fermented extract is able to inhibit the PTP1B through a synergic action between the single components of the fermented flour.

### 3.4. Molecular Dynamic Simulation for PTP1B Enzyme

**Figure 6:**
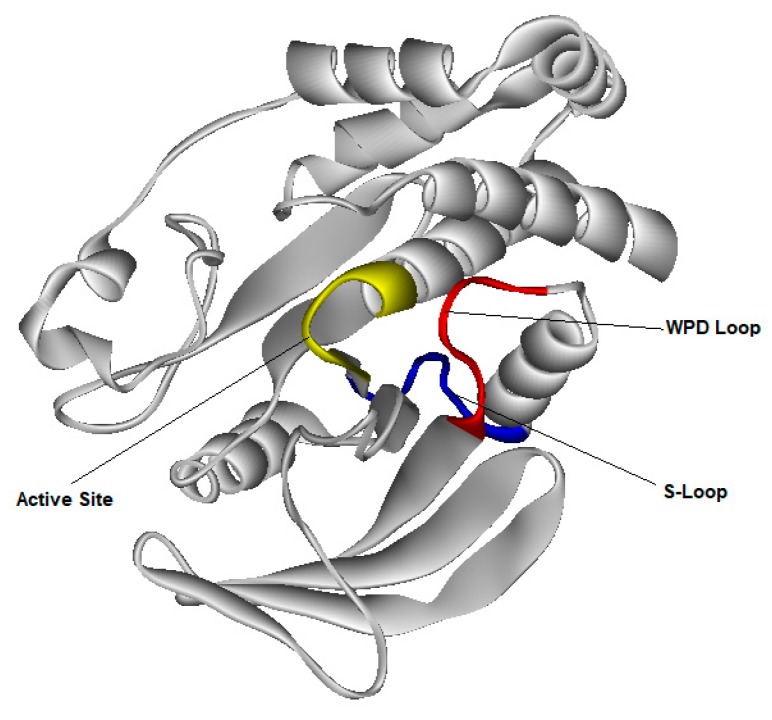
Fragment of PTB-1B structure (according to PDB ID: 1t49)**.**

Thanks to the crystallographic structure of a fragment of PTP1B (Figure 6) complexed with 3-(3,5-dibromo-4-hydroxy-benzoyl)-2-ethyl-benzofuran-6-sulfonic acid (4-sulfamoyl-phenyl)-amide allosteric inhibitor (BFS) [45], it was possible to clarify the role of WPD-Loop [46]. The structure of the complex between the benzofuran derivative (BFS) and the PTB1B fragment [45] is deposited in the Protein Data Bank (PDB ID: 1t49).

**Figure 7:**
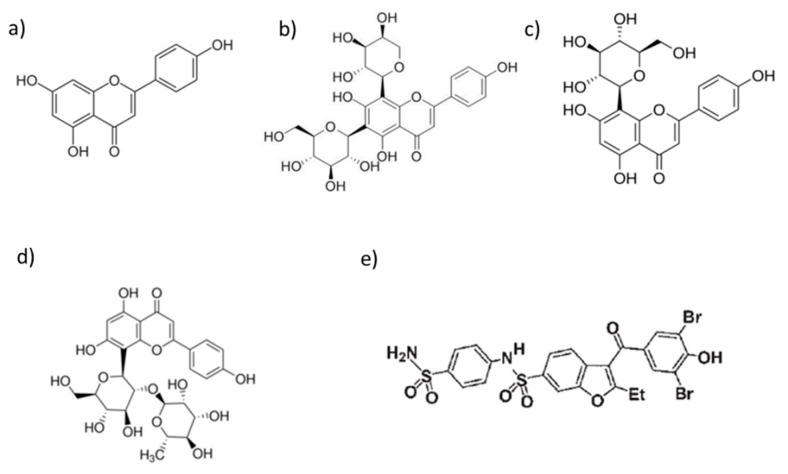
Chemical structures of Apigenin (API) (**a**), Schaftoside (SCH) (**b**), Vitexin (VIT) (**c**) Vitexin-2″-*O*-rhamnoside (VRA) (**d**) and 3-(3,5-dibromo-4-hydroxy-benzoyl)-2-ethyl-benzofuran-6-sulfonic acid(4-sulfamoyl-phenyl)-amide (BFS) (**e**).

In this work, the complex BFS-PTP1B was submitted to a MD simulation and the trajectory was compared with those obtained by complexes of PTP1B (obtained from 1t49) with four potential allosteric inhibitors with a flavonoidic structure, i.e., apigenin (API), schaftoside (SCH), vitexin (VIT) and vitexin-2″-*O*-rhamnoside (VRA) (Figure 7).

**Figure 8:**
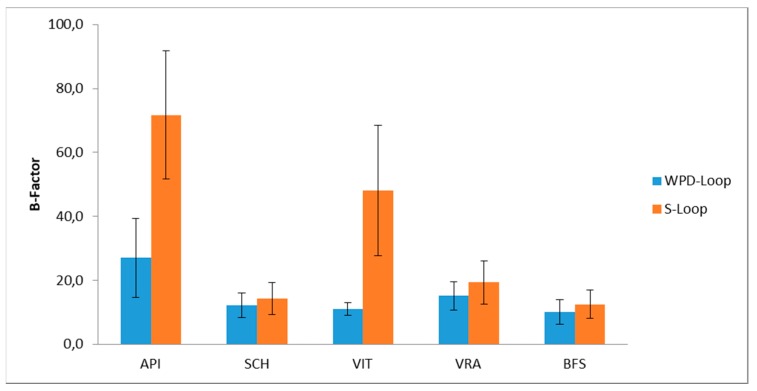
Values of B-factors calculated by the MD simulation for the complexes between PTB-1B and each of the selected flavonoids (API, SCH, VIT e VRA) and for the complex with the allosteric inhibitor BFS.

The docking position (pose) of each flavonoid with respect to the PTP-1B enzyme was determined by AUTODOCK [47]. The calculation found for all the flavonoids selected in this work, along with other possible structures of docking, the same pose of the BFS allosteric inhibitor. The MD simulation for all complexes was carried out in a vacuum at 300 K per 10ns by GROMACS package [28]. The mean mobility of WPD-Loop and of S-loop in the complex with BFS, calculated as a value of the B factor, was compared with those obtained for the complexes with the selected flavonoids, yielding the results summarized in the histogram of Figure 8. The B-factor values were calculated by the GROMACS routine [28], starting from the root mean square fluctuation (RMSF) value of alpha carbon positions in the trajectory.

As shown in Figure 8, the mean mobility of WDP-Loop in the complexes with the flavonoids SCH, VIT, and VRA was very similar to that obtained for the complex with the allosteric inhibitor BFS, while in the complex with API, the fluctuation in the WPD-Loop was significantly higher. The higher value obtained for the latter flavonoid is in agreement with the lower inhibitory activity, i.e., approximatively three times higher with respect to that of VIT [47]. The fluctuation of the WDP-Loop for VIT and its glucoside (VRA) were almost identical, suggesting that the inhibitory activity was of the same entity. Similarly, the behavior of the S-Loop in the case of API showed a significantly higher fluctuation compared to the values obtained for the other flavonoids and for the allosteric inhibitor BFS. These in silico results were in perfect agreement with the experimental results obtained for the selected flavonoids, which showed VIT and VRA with a very similar inhibitory activity of PTP1B, while API was less effective.

### 3.5. Ex Vivo Cellular Antioxidant Activity on Human Erythrocytes

**Figure 9:**
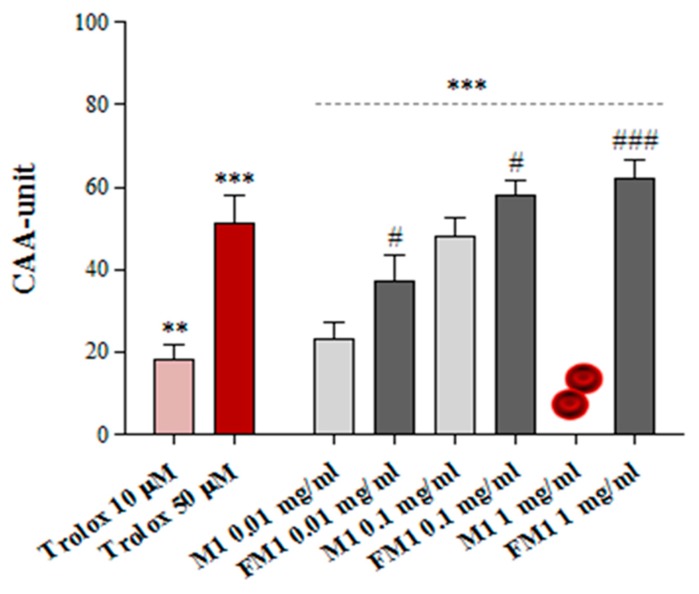
Effects of unfermented (M1) and fermented (FM1) millet extracts at different concentrations (0.01, 0.1, and 1 mg/mL) on the cellular antioxidant activity (CAA) of human erythrocytes under oxidative conditions. Trolox was used as a reference standard. Data are expressed as the mean values ± SD of independent experiments performed on three different subjects One-way ANOVA with Dunnett’s multiple comparison test: * significantly different from CNT, AAPH treated cells (CAA = 0), ** *p* ≤ 0.01, *** *p* ≤ 0.001. Unpaired t-test: ^#^ significantly different from the respective non fermented flour, # *p* ≤ 0.05, ## *p* ≤ 0.01, ### *p* ≤ 0.001.

Erythrocytes play a key role in the body in terms of anti-inflammatory and antioxidant protection, and represent a powerful tool to assess the radicals scavenging activity of many natural compounds [48]. In this study, the biological effects of millet, before and after the fermentation process, was evaluated in an ex vivo model of human erythrocytes under oxidative conditions using the CAA-RBC assay (Figure 9).

In this assay, following 1 h pretreatment with millet extracts (0.01, 0.1 and 1 mg/mL), human erythrocytes were exposed to an oxidative insult induced by the thermal decomposition of AAPH in peroxyl radicals. Our results demonstrated good, dose-dependent antioxidant protection of human erythrocytes from all tested samples with the exclusion of millet flour at the highest tested concentration (1 mg/mL), that induced erythrocyte hemolysis (Figure 9). The fermented millet obtained after 72 h of fermentation (FM1-T3) and unfermented millet (M1) extracts significantly raised the cellular antioxidant activity of human erythrocytes compared to the control, that refers to only AAPH-exposed cells (CAA = 0; ** *p* < 0.01), with CAA values being comparable to or higher than Trolox 10 and 50 μM, used as a reference standard. Additionally, the millet fermented extract exhibited, at all tested doses, significantly higher antioxidant protection than the respective unfermented ones (# *p* < 0.05).

## 4. Conclusions

This work aimed to increase knowledge about the effects of fermentation on millet. According to our results, fermentation carried out for three days using a mixture of natural microorganisms consisting mainly of *Lactobacilli* increased the total amount of phenolic compounds in the millet by more than 30%, presumably favoring the release of phenols trapped mechanically or chemically in the rate of soluble and fermentable fiber. In fact, soluble fiber has been shown to be used by *Lactobacilli* and yeasts for their growth. Furthermore, the acid hydrolysis with respect to basic hydrolysis guaranteed a higher recovery of total cinnamic derivatives and flavonoids from fermented millet, as already demonstrated for the unfermented samples. It is reasonable to say that, so far, the total phenolic content in cereals could be underestimated, due to the application of unsuitable extractive methods and due to the mechanical entrapment of these molecules by the fiber components.

Regarding the in vitro analyses, vitexin and vitexin 2″-*O*-rhamnoside partially inhibited the PTP1B enzyme, which is overexpressed in type-2 diabetes. In silico evaluations confirmed that both the main flavonoids in millet interact with WDP-Loop as allosteric inhibitors of PTP1B enzyme, suggesting that a daily intake of fermented millet may help reduce the risk of type-2 diabetes.

Our results also demonstrated a dose-dependent antioxidant protection of human erythrocytes exerted by both unfermented and fermented millet samples. According to the phenolic enrichment confirmed by chemical analyses, sourdough fermentation significantly raised the ex vivo antioxidant activities in fermented millet flour with respect to the unfermented one.

In light of our results, fermented millet, thanks to its high content of bioactive compounds, particularly flavonoids, could be proposed as a new product which is suitable for all types of consumers, and which is useful in the reformulation of recipes for celiac products for which the demand for new ingredients is constantly increasing.

## Figures and Tables

**Table 1:** List of the identified compounds in millet samples.

Analytes	[M − H]^−^	Identified Compounds
**c1**	468	N^2^,N^4^-dicaffeoylspermidine
**c2**		*p*-coumaric acid
**c5**		ferulic acid
**c8**	339	ferulic acid rhamnoside
**c9**	339	ferulic acid rhamnoside isomer
**c10**	193	isoferulic acid
**c11**	177	methylhydroxycinnamate
**c12**	192	methyl ferulate
**f3**	609	luteolin-(7-*O*-glucopyranosyl)-8-*C*-glucopyranoside
**f4**	593	vicenin II
**f6**	577	vitexin 2″-*O*-rhamnoside
**f7**	431	vitexin

**Table 2:** Free phenols and total phenols obtained after basic hydrolysis.

Free Phenols, mg/100 g				Total Phenols after Basic Hydrolysis mg/100 g		
Analytes	M1	M2	M3	M1	M2	M3
**c1**	6.8 ^a^	7.4 ^a^	7.1 ^a^	-	-	-
**c2**	-	-	-	-	-	2.3
**c5**	-	-	-	16.5 ^b^	-	34.2 ^a^
**c8**	2.5 ^a^	1.9 ^a^	1.3 ^a^	-	-	-
**c9**	3.2 ^a^	1.6 ^b^	1.6 ^b^	-	-	-
**c10**	1.1 ^a^	1.1 ^a^	0.5 ^b^	-	-	-
**c11**	1.1 ^a^	1.3 ^a^	1.2 ^a^	4.4 ^b^	-	5.3 ^a^
**c12**	1.7 ^a^	2.0 ^a^	1.1 ^b^	56.5 ^b^	40.8 ^c^	67.1 ^a^
**f3**	44.3 ^a^	22.0 ^c^	35.3 ^b^	17.3 ^a^	13.9 ^b^	3.2 ^c^
**f4**	44.7 ^a^	7.4 ^b^	7.6 ^b^	45.2 ^a^	8.6 ^b^	6.7 ^c^
**f6**	12.9 ^a^	1.5 ^c^	3.0 ^b^	-	-	-
**f7**	9.1 ^a^	2.0 ^c^	4.6 ^b^	9.4 ^a^	3.2 ^c^	4.9 ^b^
**TCC**	16.4 ^a^	15.3 ^b^	12.8 ^c^	77.4 ^b^	40.8 ^c^	108.9 ^a^
**TFC**	111 ^a^	32.9 ^c^	53.2 ^b^	71.9 ^a^	22.7 ^b^	14.8 ^c^
**TPC**	127.4 ^a^	48.2 ^c^	64.0 ^b^	149.3 ^a^	66.5 ^c^	123.7 ^b^

The data are expressed in mg/100 g of dry weight as a mean of three independent extractions. TCC: total cinnamic content; TFC: total flavonoids content; TPC: total phenolic content; RSD < 5%. Different letters (a, b and c) indicate significant differences at *p* < 0.05.

**Table 3:** Total phenolic content estimated by HPLC-DAD analysis after basic and acidic hydrolysis in the unfermented (M1) and fermented (FM1-T3) flours.

	Total Phenols after Basic Hydrolysis (mg/100 g)	Total Phenols after Acidic Hydrolysis (mg/100 g)
**M1**	149.3 ± 3.2 *	193.6 ± 4.0 *
**FM1-T3**	206.2 ± 0.8 *	278.3 ± 1.8 *

Unpaired t-test for comparing millet flours with the respective fermented flour; * *p* < 0.05.

## Supplementary Materials

The following are available online at https://www.mdpi.com/2304-8158/9/3/303/s1, Table S1: Total phenolic content in different millet varieties reported by the literature. TBC (Total Benzoic Content), TCC (Total Cinnamic Content), AF (ferulic acid content), p-CA (para coumaric acid), TPC (Total Phenolic Content). Data are expressed in µg/g.
