# Supplementary material for: Does Fermentation Really Increase the Phenolic Content in Cereals? A Study on Millet
Source: Foods. 2020 Mar 7;9(3):303. doi: 10.3390/foods9030303 (PMC7142888; doi:10.3390/foods9030303)
Supplement: Supplementary file 1 [file foods-09-00303-s001.pdf]

Table 1. Total phenolic content in different millet varieties reported by the literature. **TBC** (Total Benzoic Content), **TCC** (Total Cinnamic Content), **AF** (ferulic acid content), **p-CA** (para coumaric acid), **TPC** (Total Phenolic Content). Data are expressed in µg/g.

Millet varieties	TBC	TCC	AF	p-CA	TPC	References
Kodo	343	3429.6	2209	802	3772.6	Chandr *et al.*, 2013 [1]
Finger	156.2	416.5	358.4	41.4	572.7	Chandr *et al.*, 2013 [1]
Foxtail	174.2	1870.5	856.5	942.7	2044.7	Chandr *et al.*, 2013 [1]
Foxtail	27.8	484.2	135.2	85.1	512	Pradeep *et al.*, 2018 [2]
Foxtail	53	314.5	100	125	367.5	Pradeep *et al.*, 2018 [2]
Proso	378.4	2065.2	444.6	1235.2	2443.6	Chandr *et al.*, 2013 [1]
Little	269.3	1526.6	355.3	1085.2	1795.9	Chandr *et al.*, 2013 [1]
Little	43.7	422	128.3	175	465.7	Pradeep *et al.*, 2018 [2]
Pearl	77.2	947.4	812.3	91.9	1024.6	Chandr *et al.*, 2013 [1]

Reference

1. Chandr, A. Millet grain phenolics and their role in disease risk reduction and health promotion: A review. *J. Funct. Foods* **2013**, *5*, 570–581.
2. Pradeep, P.M.; Sreerama, Y.N. Phenolic antioxidants of foxtail and little millet cultivars and their inhibitory effects on α-amylase and α-glucosidase activities. *Food Chem.* **2018**, *247*, 46–55.
